# Iris Reconstruction: A Surgeon’s Guide

**DOI:** 10.3390/jcm13092706

**Published:** 2024-05-04

**Authors:** Lorenzo Ferro Desideri, Kirupakaran Arun, Grace Doherty, Enrico Bernardi, Rodrigo Anguita

**Affiliations:** 1 Department of Ophthalmology, Inselspital, Bern University Hospital, University of Bern, Freiburgstrasse 15, CH-3010 Bern, Switzerland; lorenzoferrodes@gmail.com (L.F.D.); enrico.bernardi@insel.ch (E.B.); 2 Department for BioMedical Research, University of Bern, Murtenstrasse 24, CH-3008 Bern, Switzerland; 3 Bern Photographic Reading Center, Inselspital, Bern University Hospital, University of Bern, CH-3010 Bern, Switzerland; 4 Moorfields Eye Hospital, NHS Foundation Trust, London EC1V 2PD, UK; kirupakaran.arun@nhs.net; 5 Faculty of Medicine, Queen’s University Belfast School of Medicine, Belfast BT9 7BL, UK; gdoherty34@qub.ac.uk

**Keywords:** iris reconstruction, iris defects, prosthetic iris devices, iridodialysis repair, iris surgery

## Abstract

**Objectives**: The aim of this review paper is to summarise surgical options available for repairing iris defects at the iris–lens plane, focusing on suturing techniques, iridodialysis repair, and prosthetic iris devices. **Methods**: A thorough literature search was conducted using multiple databases, including Medline, PubMed, Web of Science Core Collection, and the Cochrane Library, from inception to February 2024. Relevant studies were screened based on predefined criteria, and primary references cited in selected articles were also reviewed. **Results**: Various surgical techniques were identified for iris defect repair. Suturing methods such as interrupted full-thickness sutures and the McCannel technique offer solutions for smaller defects, while iridodialysis repair techniques address detachment of the iris from the ciliary body. Prosthetic iris devices, including iris–lens diaphragm devices, endocapsular capsular tension ring-based devices, and customizable artificial iris implants, provide options for larger defects, each with its own advantages and limitations. **Conclusions**: Successful iris reconstruction requires a personalised approach considering factors like defect size, ocular comorbidities, and patient preference. Surgeons must possess a thorough understanding of available techniques and prosthetic devices to achieve optimal outcomes in terms of both visual function and, nonetheless, cosmetic appearance.

## 1. Introduction

The natural iris plays an essential role in controlling the diameter of the pupil and therefore modulates the amount of light reaching the retina. It consists of two layers: the anterior fibrovascular iris stroma and the posterior pigmented iris pigment epithelium. A healthy iris diaphragm plays an essential role in minimising glare, photophobia, and higher-order aberrations from the lens and peripheral cornea [1,2,3].

Iris defects can be classified as congenital or acquired. Acquired causes can result from penetrating injuries, iatrogenic damage such as during phacoemulsification or iridectomy, and oncological removal of tumour lesions [4]. The extent of the defect varies greatly, from a few hours of iris absence to near total or complete loss. Regardless of the cause, iris defects can cause significant cosmetic concerns as well as functional implications such as reduced visual quality, severe glare, and loss of contrast sensitivity [5,6]. 

The management of iris defects depends on the size of the defect, other ocular comorbidities, ocular anatomy such as capsular stability and lens status, and the patient’s preference for cosmesis. Iris replacement can be achieved at the ocular surface with coloured contact lenses, at the corneal level with corneal tattooing, in the anterior chamber with intraocular lenses with coloured iris diaphragm, and at the iris level with sutures and iris prosthetic devices. However, it is important to note that, while iris replacement options such as intraocular lenses with coloured iris diaphragms were previously considered for placement in the anterior chamber, medical contraindications make these options impractical and thus inviable options; furthermore, there are not approved treatments [7,8,9].

In this review, we aim to summarise the current surgical options available to repair an iris defect at the iris–lens plane, describe the surgical technique required and complications, and evaluate surgical outcomes.

### Materials and Methods

A comprehensive literature search was conducted to identify all published studies on these topics from database inception to February 2024. The following databases were searched, i.e., Medline, PubMed, Web of Science Core Collection, and the Cochrane Library. The following words “iris, iris reconstruction, surgical iris reconstruction, trauma, aniridia” with and without terms such as AND/OR were used, yielding a total of 246 articles. Languages other than English were excluded. The remaining abstracts were screened and included based on their relevance to the review article. This was decided on an individual basis after reviewing each of these articles. In addition, the primary references cited in the articles were also reviewed.

## 2. Techniques

There are several techniques available for iris repair. These approaches have undergone significant changes over the past 25 years as we have come to understand the significance of pupil size and regularity of pupil aperture in achieving optimal visual outcomes.

Small iris defects (less than 2–3 clock hours) can be managed with suturing the residual iris margins together. Iris defects that are larger than 3 clock hours are not suitable for surgical closure and require a prosthetic iris device. 

### 2.1. Suturing Iris Defects/Pupilloplasty

One of the first methods used to repair an iris defect was through suturing. This is commonly known as a pupilloplasty, and there are various approaches that have been demonstrated over the years. They all work in slightly different ways with the aim of creating a round pupil and closing the iris defect. The major disadvantage of the pupilloplasty approach is that further iris damage can occur from intraoperative manipulation and there may not be sufficient reduction in pupil aperture. One important aspect to keep in mind before performing a pupilloplasty is to remove any vitreous in the anterior chamber. This is important for ensuring clear visualisation, minimizing complications, optimizing outcomes, and promoting a smoother post-operative recovery for a patient [10]. 

### 2.2. Interrupted Full-Thickness Sutures to Bring Iris Margins Together

Small defects, such as those under 2 clock hours, can be closed by applying 2–3 full-thickness sutures through the iris sphincter of the unopposed iris margins [11]. The first suture should ideally be placed close to the margin of the defect in order to prevent iris tenting [12].

### 2.3. McCannel Technique

Slightly larger iris defects (2–3 clock hours) often require different approaches to successfully appose the 2 edges of the iris defect. McCannel first described a technique in 1976 when trying to repair an iris defect following complicated intracapsular cataract extraction [13]. In this technique, a long needle with a 10/0 polypropylene suture was passed through a limbal paracentesis. The needle is then passed through the 2 edges of the iris defect and is taken out of the anterior chamber through another limbal paracentesis before being cut. Viscoelastic is injected in the anterior chamber below the iris to help direct the iris edges towards the suture. A Sinskey hook is then passed through a stab incision (made at the limbus between the 2 paracentesis sites) to bring both ends of the suture out of the anterior chamber to be tied (pulling the edges of the iris defect together) and cut. This process is repeated until the iris defect it closed.

### 2.4. Shin Technique

Shin modified McCannel’s technique by using a 1.6 cm 25-gauge hypodermic needle attached to a tuberculin syringe [14]. The needle is passed through 1 limbal paracentesis, and then it pierces both the proximal and distal iris margins from anterior to posterior and is then taken out form an opposite limbal paracentesis. The 25-gaude needle is then removed, and the 10/0 polypropylene suture is removed through the stab incision with the help of a Sinskey hook. The suture ends are then tied, cut, and buried.

The technique was further modified by Siepser in 1994 to use 2 paracenteses and a slipknot [15]. The 10/0 propylene suture is introduced through an initial paracentesis, passed through the iris defect margins, and brought out through the opposite paracentesis. A loop is then formed with a suture (1st suture) and withdrawn with a Sinskey hook through the opposite paracentesis while holding the end of this suture, thereby bypassing the anterior chamber [16]. A slipknot is then tied extraocularly to the other suture (2nd suture) so that the sutures are extracted outwards, the slipknot is pulled into the anterior chamber, and the iris edges are apposed together [17]. The suture ends are then trimmed, and this process is repeated until the iris defect is fully closed.

The most modern variation in the pupilloplasty procedure is Dr Agarwal’s single four-throw pupilloplasty [18]. In this technique, a needle is passed through the proximal and distal portions of the iris tissue until they are approximated, and then, a loop of suture is withdrawn. The suture end is passed through the loop 4 times. When the suture ends are pulled, the loop slides inside the iris tissue. It provides a self-retaining knot and is more time-consuming that previous pupilloplasty techniques [19].

Another technique that may be used in conjunction or separately to closing an iris defect is pupil cerclage [16,20,21]. This technique is used when a smaller pupillary aperture is required and involves a running suture being passed around the pupillary margin to create a purse-string suture. A long, curved 10/0 polypropylene needle is passed in and out of the anterior chamber through limbal paracenteses while the needle is weaved through the iris near the pupillary margin to form the cerclage. A modified version using a slipknot allows the same cerclage whilst reducing the risk of iatrogenic iris damage [21]. 

## 3. Iridodialysis Repair

Iridodialysis is a separation of the iris from its attachment to the ciliary body and most commonly occurs secondary to blunt trauma, penetrating trauma, and complicated intraocular surgery due to the thin and weak structure of the iris root [22]. Cases of small iridodialysis or superior iridodialysis that is covered by the upper lid are often asymptomatic. However, other cases tend to cause visual problems such as glare, diplopia, and photophobia and as such require surgical intervention [23]. 

A number of surgical techniques have been described for repair of iridodialysis. They can be classified as open chamber or closed chamber techniques [24]. Open chamber techniques access the site of iridodialysis through a limbal self-sealing incision (such as the phacoemulsification incision) or a scleral tunnel incision. Paton first described the open chamber approach for iridodialysis repair, but this has now become obsolete [25]. Closed-chamber techniques gain access to the anterior chamber with a needle [26], and there are numerous variations as described below. Prior to surgery, miotic agents should be used to shrink the pupil and thus stretch and increase the surface area of the iris. 

### 3.1. McCannel Technique of Iridodialysis Repair

**Figure 1:**
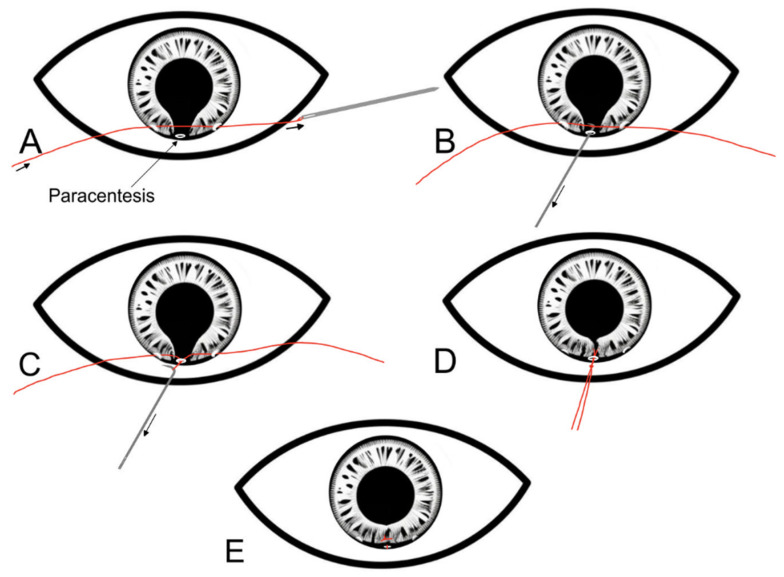
Step-by-step diagrammatic representation of the McCannel technique for repairing an iris defect. (**A**) Two limbal paracenteses are made 90 degrees away from the iris defect. A long needle with a 10/0 polypropylene suture is passed through 1 paracetesis, the edges of the iris defect, and then through the other paracentesis. (**B**) A Sinskey hook is introduced through a limbal paracentesis at the site of the iris defect. (**C**) The Sinskey hook is used to bring both ends of the suture out of the anterior chamber. (**D**) The suture is securely tied with a knot outside the eye. (**E**) After the knot is secure, it is slipped into the anterior chamber and secured over the iris.

This technique uses two double-armed sutures and aims to fixate the peripheral iris to the scleral wall approximately 1 mm posterior to the limbus [27]. This technique begins with one needle entering the anterior chamber through the limbus inferiorly. It is then guided to pierce the iris base and leave the anterior chamber angle and sclera. A second needle is then used in a similar way and enters via the same incision and pierces the iris base adjacent to the previous needle. The suture is then tied over the sclera and buried. This technique requires less manipulation than other techniques of iridodialysis repair; however, one potential complication is the erosion of the suture through the superficial layers of the eye [23] (Figure 1).

### 3.2. Hangback Technique of Iridodialysis Repair

The hangback technique works best for cases of iridodialysis that are less than 3 clock hours in size and uses a 10/0 polypropylene suture to suspend the detached iris root to the normal point of iris insertion [28]. A partial thickness scleral tunnel is created parallel to the iridodialysis and 2 mm posterior to the limbus and a side port is created 3–4 clock hours away. The needle is inserted through the paracentesis and then passed through the iris root and then leaves the anterior chamber via the scleral tunnel. The second arm of the suture is then passed similarly, and this time through the iris root adjacent to the initial suture arm. A horizontal mattress suture is placed ab interno and tightened to bring the iris periphery just under the limus, and this knot remains in the anterior chamber. 

### 3.3. Sewing Machine Technique of Iridodialysis Repair

**Figure 2:**
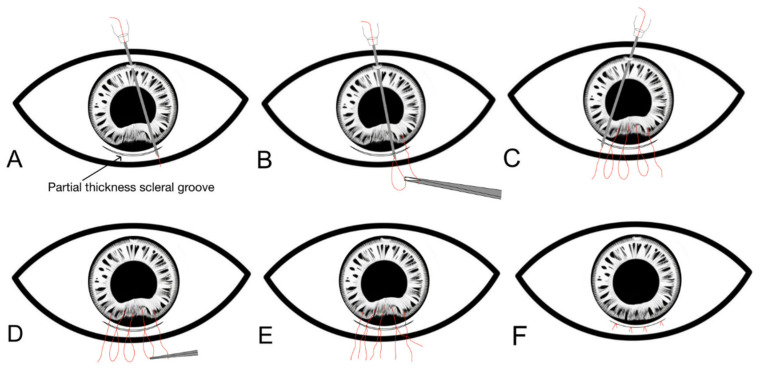
Step-by-step diagrammatic representation of the sewing machine technique of iridodialysis repair. (**A**) A scleral tunnel is created parallel to the dialysis, and a paracentesis is made opposite to the dialysis. A 10/0 prolene suture is threaded into a long 26-guage needle, and this is passed through a paracentesis, and then through the torn iris root, and it exits through a partial thickness scleral groove. (**B**) The free end of the suture is pulled out through the scleral tunnel, and the first suture loop is created outside the scleral tunnel. (**C**) Creation of suture loops all along the dialysis by passing the pre-threaded 26-gauge needle with the suture through the root of the iris dialysis and scleral tunnel from inside out. (**D**) The suture loops are then cut. (**E**) Adjacent ends of the suture loops are tied to each other. (**F**) The suture knots should be buried into the scleral tunnel with the overlying conjunctiva closed.

A 10/0 polypropylene suture is threaded into a long 26-gauge needle [29]. A scleral tunnel is made parallel to the iridodialysis and 2 mm posterior to the limbus and a side port is created opposite to the dialysis. The needle with the suture is passed through the paracentesis, and then through the torn iris root, and it exits the anterior chamber through the scleral groove. The free end of the suture is then pulled out through the scleral tunnel, and a suture loop is created by withdrawing the needle in the anterior chamber with an assistant holding onto the exteriorised suture. This process is repeated to create multiple suture loops all along the dialysis. The suture loops are then cut, and the adjacent ends are then tied with the knots buried into the scleral tunnel (Figure 2).

### 3.4. Cobbler’s Technique of Iridodialysis Repair

In this technique, a partial thickness scleral tunnel is created parallel and 1.5 mm behind the limbus along the extent of the iridodialysis [30]. A limbal paracentesis is created opposite to the scleral tunnel. A 10/0 polypropylene suture is threaded through a 26-guage needle, and then, this needle enters the paracentesis, engages the iris root, and exits through the scleral groove. The free end of the prolene suture is pulled out. The needle is withdrawn into the anterior chamber to engage the iris root again. The suture is pulled out forming a loop through which the free end of the suture is passed to lock the loop. This step is repeated multiple times so that multiple loops are laid over the scleral bed, and then, the loops are tied. 

## 4. Prosthetic Iris Devices

Since the first iris–lens plane implant was used in 1991 [10], there have been numerous technological advancements in the field of prosthetic iris devices. The choice of which prosthetic iris device to use should be individualised based on the ocular anatomy and patient’s choice.

Numerous prosthetic iris devices are available for iris defects that are larger than 3 clock hours in size. 

Prosthetic iris devices can be categorised into three main designs:

1. Iris–lens diaphragm devices;
2. Endocaspular capsular tension ring-based devices;
3. Customizable artificial iris.

Prosthetic iris devices are available in a wide range of models, offering variations in fixation methods, placement locations, sizes, materials, and costs. Surgeons can select the most suitable option for each patient, considering their unique anatomical factors. The severity and extent of the iris defect, the status of the intraocular lens, and the presence or absence of an intact capsular bag are crucial determinants in choosing the appropriate device. Furthermore, it is important to consider that patients may have different levels of concerns regarding post-operative cosmetic appearance and symmetry with their other eye. Surgeons should carefully evaluate these anatomical and cosmetic factors to make an informed decision and provide the patient with the most appropriate prosthetic iris device.

### 4.1. Iris–Lens Diaphragm Devices

The first posterior chamber prosthetic iris device was inserted in 1991 by Sundmacher [10,31]. These lenses consist of a rigid iris–lens diaphragm with a peripheral opaque annulus and IOL style haptics that function as an artificial iris. They have a large diameter and a very rigid structure as they are made of polymethylmethacrylate (PMMA). Due to their size and rigid structure, they require a large 10.5 mm limbal incision. Depending on if there is adequate capsular support, iris–lens diaphragm lenses can be placed passively in the sulcus or sutured to the scleral through fixation loops on the haptics of the lenses [32,33,34].

If there is a plan for transscleral suture fixation, a conjunctival peritomy is created along the superior limbus from 10 to 2 o’clock. The 10 mm wide superior limbal incision is made with scleral flaps at 2 and 8 o clock for transscleral fixation. Once the anterior chamber is filled with viscoelastic, the iris–lens diaphragm device is injected into the anterior chamber, and transscleral fixation is performed 1.5 mm posterior to the limbus at the level of the sulcus using 9/0 prolene [35,36,37]. If there is enough capsular support to allow passive sulcus implantation, then, there is no need for a conjunctival peritomy, but a large 10 mm limbal incision is still required. Viscoelastic should be used to lift away the iris remnants, and care should be taken to ensure the device is implanted in the sulcus instead of anterior to the iris or in the anterior chamber angle.

The only currently available option for rigid prosthetic iris–lens diaphragm implant is the Morcher 67B implant. This implant consists of a 12.5 mm aniridia implant with a 3 mm pupil aperture [38]. Various pupil apertures (3.5 mm to 6.5 mm) are available in different models. Having the option of a 3 mm pupil size can help greatly in patients complaining of significant photophobia [39]. One of the major disadvantages of the Morcher 67 implant is that there are only plain black artificial iris options, so it offers no chance of cosmetic symmetry [40]. More recently, Morcher have developed models that do come in 45 different colours [41]. However, this device is no longer available due to failing to meet the latest Medical Devices Regulation (MDR) Standards.

The main advantage of using iris–lens diaphragm devices is their ability to correct both a large iris defect combined with a phakia with a simple implant. Studies have shown that patients report improvement in visual acuity and significant reduction (75–100%) in glare following implantation [42,43,44]. However, they also have various disadvantages. Due to their large size and rigid material, they all require a large corneal incision varying between 8 and 11 mm for insertion. This inevitably leads to considerable induced astigmatism. In addition, there are a few reported intra-operative complications associated with iris–lens diaphragm devices, including broken implants during implantation, which is thought to be due to the brittleness of the PMMA material [20]. In 20–30% of cases, implantation at a site other than the sulcus has been observed, which can lead to decentration, migration, and tilting of the device. This was more commonly witnessed in cases where transscleral suture fixation was required [45]. Iris–lens diaphragm devices, due to their large size, can be challenging to rotate and manoeuvre in the anterior chamber. Consequently, they are associated with a higher risk of iatrogenic intra-operative damage to local structures and complications such as further iris damage, uveitis–glaucoma–hyphaema syndrome, and corneal decompensation [46]. 

### 4.2. Endocapsular Capsular Tension Ring-Based Devices

Endocapsular capsular tension ring (CTR)-based devices comprise a CTR backbone with various segmental iris plates. These devices are much smaller than the rigid iris–diaphragm devices and as such can be inserted through a 3.5 mm wound. They are usually inserted into the capsule but can also be inserted into the sulcus, but both positions require an intact capsulorrhexis and good capsular support. If the endocapsular CTR devices are inserted in conjunction with cataract surgery, the IOL should be implanted first. This is because the sharp edges of the CTR device are more likely to rupture the capsule if it is inserted before the IOL [35]. The capsule should then be stained (if not already performed) as the opaque iris lamellae block the red reflex. Trypan blue is most commonly used, but some authors report that indocyanine green is more useful as it does not reduce capsular elasticity and also fluoresces in the red spectrum under the operating microscope [47,48]. Viscoelastic should be used to open the potential space between the IOL and the anterior capsule before the device is dialled in the capsular bag. 

Morcher produces two different types of rings: the Type 50 endocapsular CTR aniridia rings are used for complete iris loss and the Type 96 aniridia rings are for segmental iris loss (Figure 1). The Morcher 50 model rings feature fins of varying widths, enabling the creation of pupil sizes ranging from 3.5 mm to 6.5 mm. Comprising eight fins, these rings are implanted sequentially atop each other and the capsular bag. Subsequently, they are carefully dilated to ensure interdigitation of the segments, achieving comprehensive peripheral iris coverage. The Morcher 96 model rings consist of single fin segments and vary in size between 90 and 180°. Larger iris defects can be managed by using two devices and by positioning them side by side.

Another endocapsular CTR device is from Ophtec BV. It consists of two rectangular iris segments and a central locking element. Once placed in the capsular bag, considerable intraocular dexterity and manipulation are required to lock the fixation and elements in place [49]. However, this device is no longer available due to failing to meet MDR standards.

The main advantages of the endocapsular CTR devices are that they can be inserted with a small corneal incision, which reduces post-operative astigmatism [48]. Furthermore, the option of placement of this device within the capsular bag has been proven to minimise post-operative inflammation and the incidence of uveitis–glaucoma–hyphaema syndrome [50]. 

The main complication with these devices is that, when the correct alignment of two devices is required, it can be technically challenging, and there are reports that these devices can fracture if over-manipulated [42]. However, the more recent 50F variants have narrower gaps between the fins to make the alignment process far easier and less time-consuming due to the need for less intraocular manipulation [40]. 

### 4.3. Customizable Artificial Iris

The customisable artificial iris implant consists of only the iris diaphragm with no central optic, providing a cosmetic appearance that is far superior to what is possible with iris diaphragm devices or an iris ring. The customisable artificial iris is designed individually for each patient from a photograph of the contralateral healthy iris of the fellow eye [35]. The absolute contraindication for this type of implant is the absence of prior cataract surgery. The implant itself has been demonstrated to precipitate cataract formation. Moreover, due to the implant’s placement and fixed pupil aperture size, subsequent cataract removal becomes inaccessible unless the implant is first extracted. Therefore, cataract surgery must be performed before implantation to ensure proper access for potential future interventions related to the cataract [51]. 

TheHumanOptics CUSTOMFLEX(Erlangen, Germany) (Artificial Iris is the most commonly used type of customised artificial iris and has been available since 2002 [52]. It is made of flexible biocompatible hydrophobic silicone elastomer, which is the same material used in HumanOptics silicone intraocular lenses and has passed biocompatibility tests in accordance with International Standard Organization [53]. It is steam sterilised and is packaged in a sealed container filled with sterile saline The implant has a 12.8 mm diameter and a fixed pupil size of 3.35 mm. Prior to implantation, the iris is cut to the desired diameter for the patient using a corneal trephine. It has an extremely thin profile of 0.4 mm at the centre and 0.25 mm at the periphery. It is available in two formats, with or without internal fibres. The “with fibre” design includes an embedded polyester mesh that provides enhanced rigidity to facilitate suturing of the implant, for example, to residual iris tissue, to sclera, or to an IOL. The “fibre-free” design is often used for passive implantation into the sulcus or capsular bag. The silicone material makes the implant flexible, which has many advantages. The incision size required varies from 2.5 mm to 3.2 mm, depending on whether internal fibres have been incorporated. In addition, the implant itself can be implanted in a number of ways [52]. It can be inserted via an IOL injector or manually folded and inserted with forceps. If the capsular bag is stable, it can be implanted directly into the capsular bag accompanied with an IOL. If there is inadequate capsular support, then, it can be positioned with transscleral fixation (similar to iris–diaphragm approaches). 

A more novel approach that has been used with these implants in patients who are aphakic is the “sandwich technique” where a three-piece IOL is fixed to the back of the artificial iris implant using the IOL haptics [54]. Penetration sites (for the IOL haptics) are marked 1 mm away from the outer iris implant rim at 0 and 180 degrees. A bent 30-gauge cannula is then pierced through the artificial iris at the marked penetration sites, and the haptics are inserted into the cannula so that the haptic ends eventually lie on top of the anterior surface of the artificial iris and the optic remains posterior to the artificial iris [8]. 

Aside from the cosmetic benefits of the HumanOptics Artifical Iris implant, studies have also shown 94% of patients rated the cosmesis as improved, much improved, or very much improved (assessed with Global Aesthetic Improvement Scale). Patients reported a subjective decrease at 12 months in light sensitivity in the daytime (60%), light sensitivity at night (42%), glare during the day (53%), and glare at night (49%) [55,56]. Although, the CUSTOMFLEX Artificial Iris implant is not designed to improve vision, 67% of eye had better uncorrected visual acuity following surgery and 28% had unchanged visual acuity at 12 months [56,57]. 

One of the disadvantages of this implant is that the pupil aperture is fixed, and symmetry of the other eye can only be achieved in medium light intensity, so it is important to manage patient expectations for this. One of the factors that appears to play a big role in cosmetic outcome is centration of the artificial pupil, so it is important for surgeons to bear this in mind intra-operatively [58]. The post-operative complication rates were low but did include hypotony, raised IOP, dislocation of the implant, and post-operative iritis. As with the iris–lens diaphragm devices, a higher rate of dislocation, and need for further surgery to reposition, the device was used when suture fixation was required. One study has demonstrated that adjusting the artificial iris size with trephination does reduce the need for repositioning as a result of migration [59].

## 5. Conclusions

Surgical iris reconstruction requires a personalised approach based on the extent and nature of the iris defect. Minor defects, such as those spanning less than 2 clock hours or involving tears at the iris base, can typically be addressed through suturing alone. However, larger defects or complete iris tissue loss requires the use of a prosthetic iris device.

There are three main designs of prosthetic iris devices available: iris–lens diaphragm devices, endocapsular capsular tension ring (CTR)-based devices, and customizable artificial iris implants. Each design has its own unique features, advantages, and limitations. It is crucial to have a comprehensive understanding of these factors when selecting the most appropriate device for a specific patient. To achieve optimal outcomes and ensure the successful reconstruction of the iris, while preserving or restoring the patient’s visual function and aesthetic appearance, surgeons must possess a thorough knowledge of various prosthetic iris device models, their surgical implantation techniques, and their respective strengths and weaknesses.

## Data Availability

No applicable.
